# Supplementary material for: Exploring a model-based analysis of patient derived xenograft studies in oncology drug development
Source: PeerJ. 2021 Jan 27;9:e10681. doi: 10.7717/peerj.10681 (PMC7847196; doi:10.7717/peerj.10681)
Supplement: Supplemental Information 2 [file peerj-09-10681-s002.docx]

## Derivation of linear growth law

Assume that the tumour is spherical with radius $r$, if the proliferating layer has thickness $d<r$, and is growing at a rate a, then the volume of the layer is

$$V_{p}=4\pi r^{2}d$$

and it is growing at a rate

$\frac{dV_{p}}{dt}=aV_{p}=a4\pi r^{2}d$.

The total volume of the tumour is

$$V=V_{p}+V_{c}$$

where Vc is the volume of the necrotic core. The growth equation for the radius of the whole tumour is given by

$$\frac{dr}{dt}=\frac{dr}{dV}\frac{dV}{dt}=\frac{dr}{dV}\left( \frac{dV_{p}}{dt}+\frac{dV_{c}}{dt} \right)$$

but since

$$\frac{dV_{c}}{dt}=0$$

the growth equation of the radius becomes

$$\frac{dr}{dt}=\frac{dr}{dV}\frac{dV}{dt}=\frac{dr}{dV}\frac{dV_{p}}{dt}=\left( \frac{1}{4\pi r^{2}} \right)a4\pi r^{2}d=ad$$

which is solved to give the linear equation

$r=R_{0}+adt$.

## Analysis of PDX controls

We found that using a proportional error model gave a better model fit to the control data truncated at 14 days than using an additive model (-2xlog-likleihood – proportional error = 668; -2xlog-likleihood – additive error = 750). The final model parameters for the control fit can be seen in Table S1, these values were used for the PDX simulation study. A posterior (visual) predictive check can be seen in Figure S1.

Table S1

Figure S1

Solid dots are the observed values, solid red-line is the observed median over time, solid blue lines are the observed 2.5^th^ and 97.5^th^ percentiles over time, the pink shaded region is the 95% confidence interval for the median and the blue shaded regions are the 95% confidence intervals for the 2.5^th^ and 97.5^th^ percentile both derived by simulation.

We found that modelling the volume of the tumour as opposed to the radius resulted in little change to the power of the analysis. Figure S2 shows the results of the 14, 21 and 28 day 2-arm, treatment versus control, study analysis of the PDX data when the regression and t-test were both performed on the tumour volume

Figure S2

We also compared mixed-effect modelling to a naïve pooled approach. Figure S3 shows the comparison of the statistical power. There was a noticeable reduction in power if a naïve pooled approach was taken instead of using a mixed-effects model if the study endpoint was 14 days, but there was little difference in power when the length of study endpoint was day 21 or 28.

Figure S3

## Analysis of CDX controls

The final model parameters to the control data, using a proportional error model, in the CDX study can be seen in Table S2, these values were used in the CDX simulation study. A posterior (visual) predictive check can be seen in Figure S4.

Table S2

Figure S4

Solid dots are the observed values, solid red-line is the observed median over time, solid blue lines are the observed 2.5^th^ and 97.5^th^ percentiles over time, the pink shaded region is the 95% confidence interval for the median and the blue shaded regions are the 95% confidence intervals for the 2.5^th^ and 97.5^th^ percentile both derived by simulation.

## Analysis of PDX studies

For each study, the TGI and treatment effect parameter, calculated by the model-based approach, are reported in Table S3.

Table S3
