# Supplementary figures and images for: Exploring a model-based analysis of patient derived xenograft studies in oncology drug development
Source: PeerJ. 2021 Jan 27;9:e10681. doi: 10.7717/peerj.10681 (PMC7847196; doi:10.7717/peerj.10681)

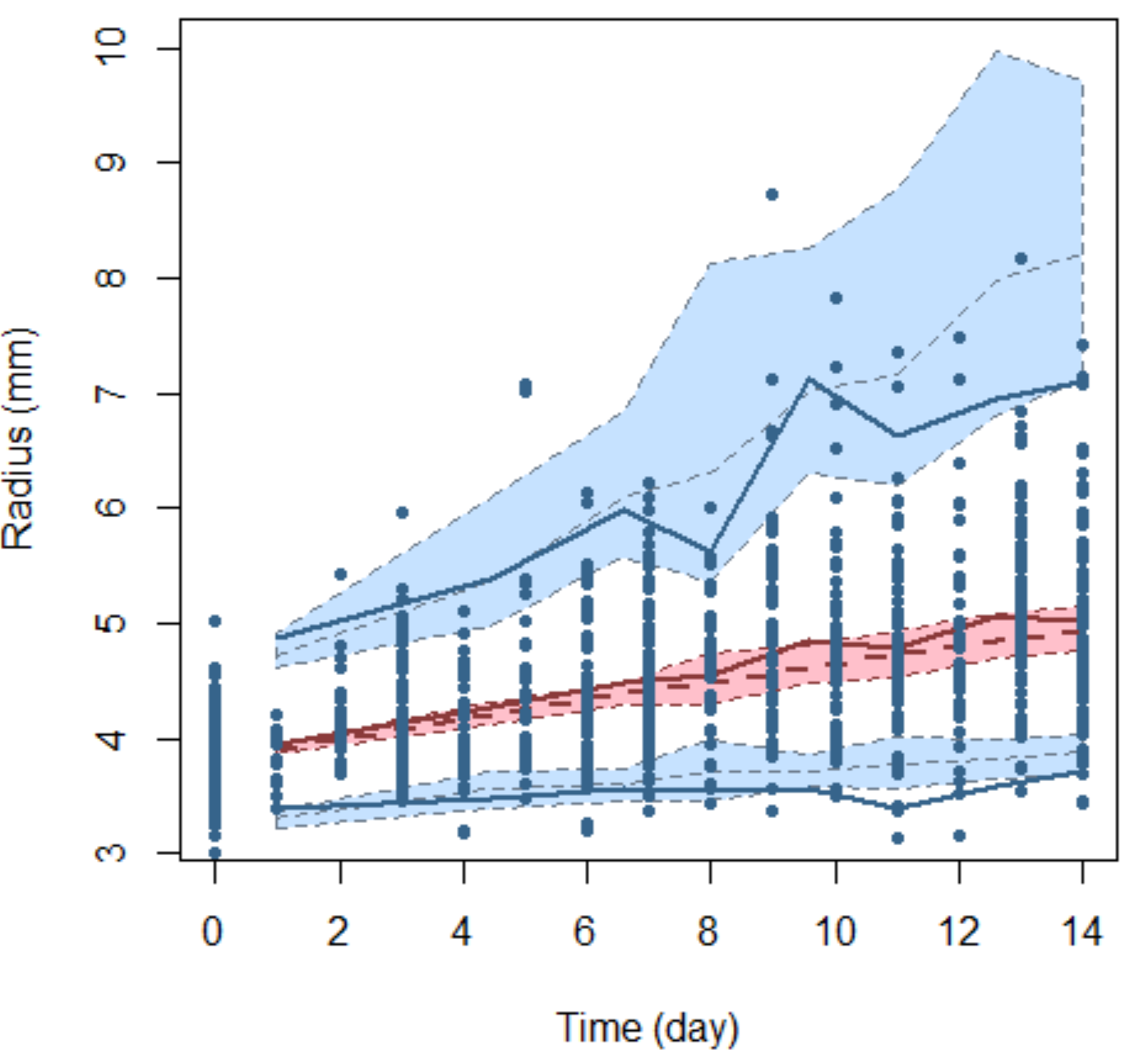

Supplement: Figure S1 [file peerj-09-10681-s003.png]

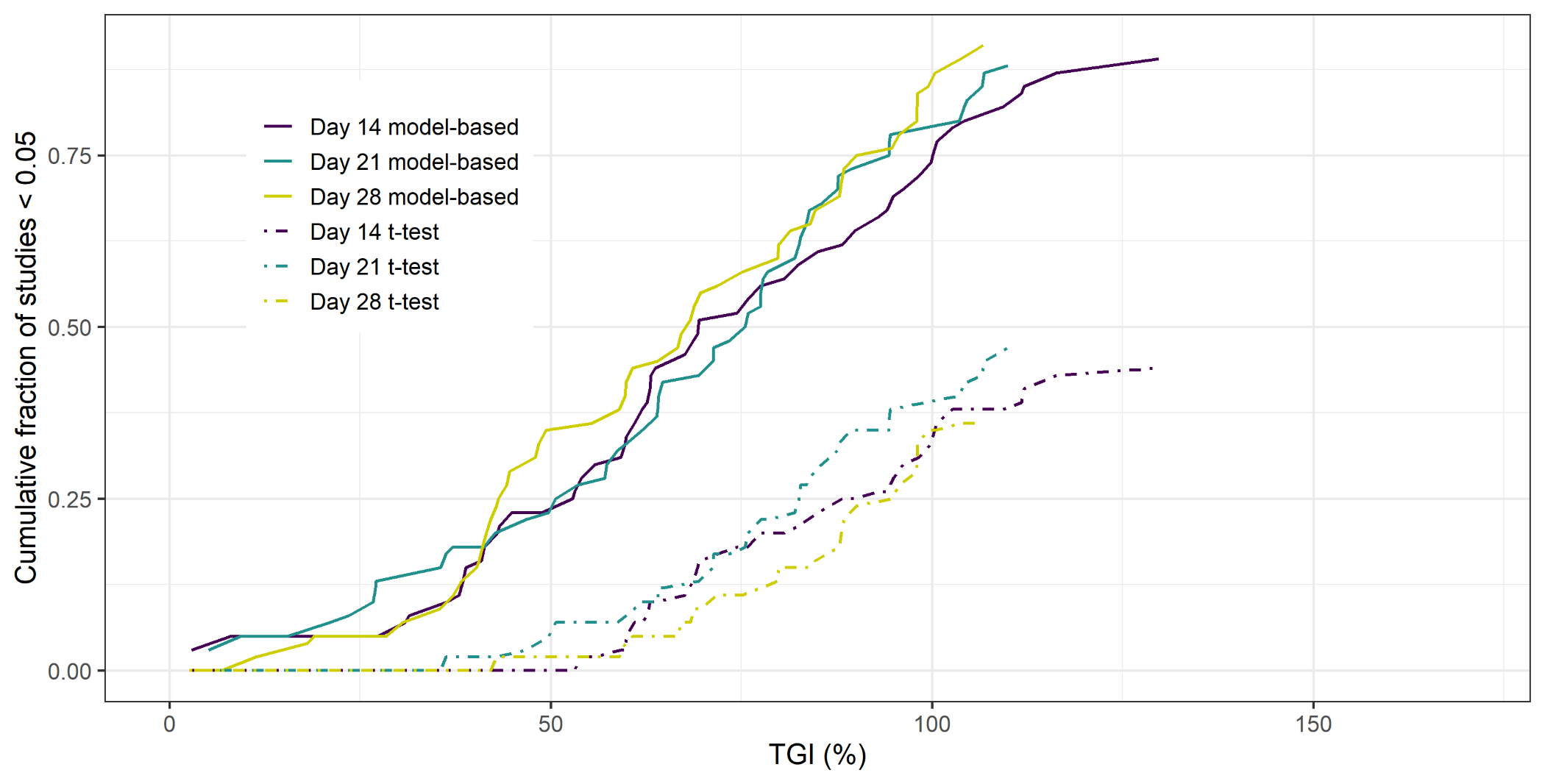

Supplement: Figure S2 [file peerj-09-10681-s004.png]

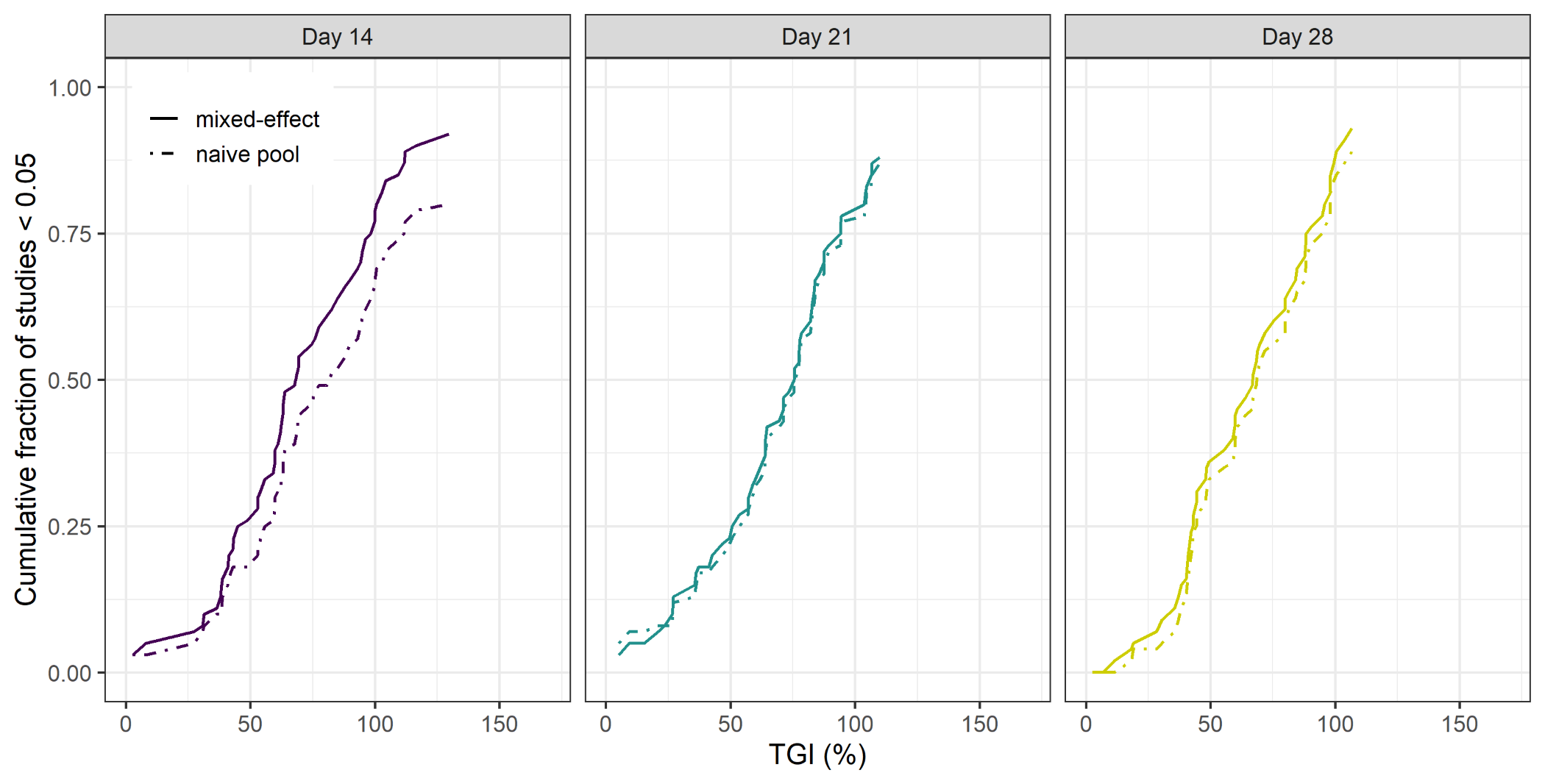

Supplement: Figure S3 [file peerj-09-10681-s005.png]

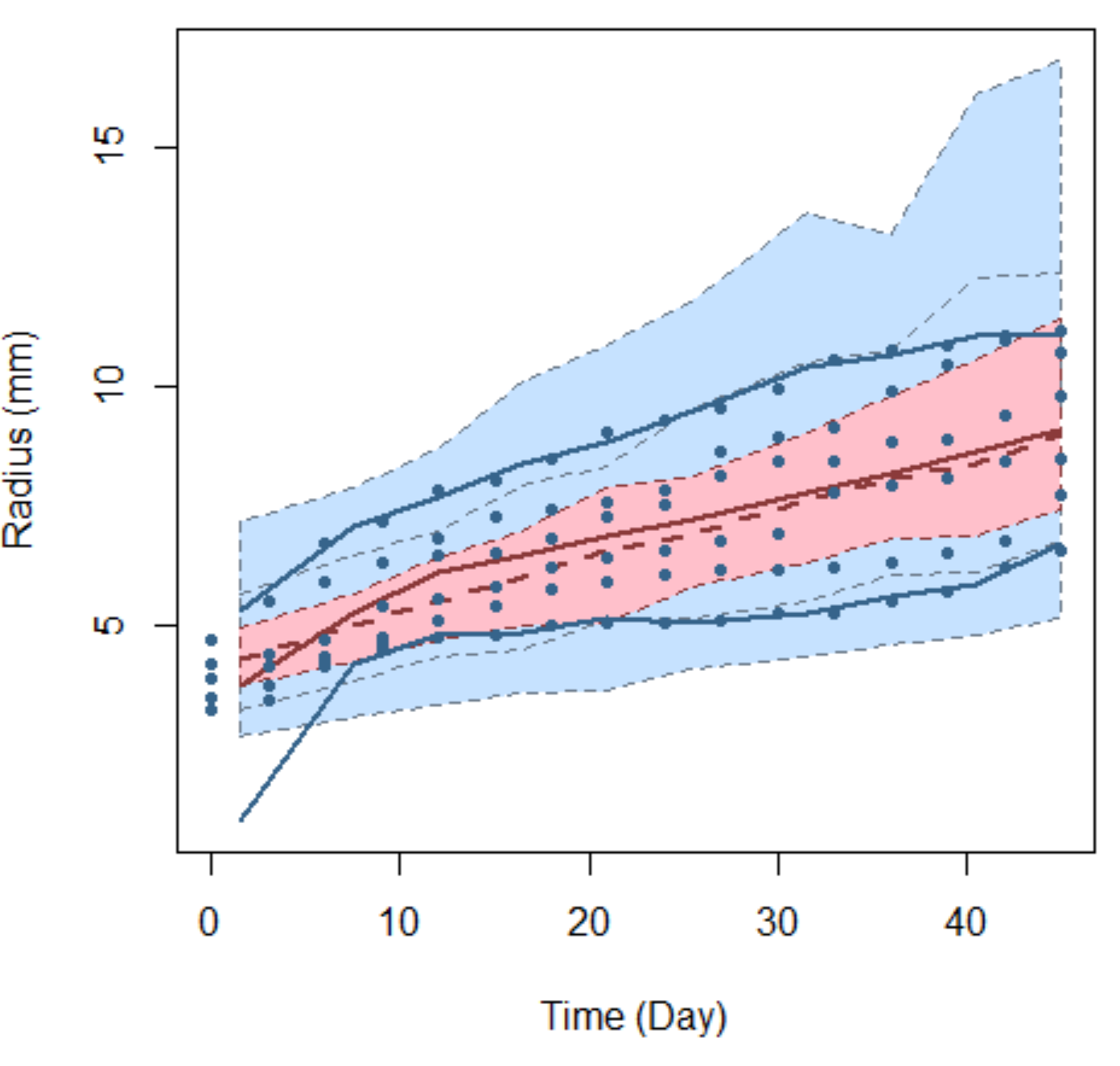

Supplement: Figure S4 [file peerj-09-10681-s006.png]
