# Supplementary material for: Exploring a model-based analysis of patient derived xenograft studies in oncology drug development
Source: PeerJ. 2021 Jan 27;9:e10681. doi: 10.7717/peerj.10681 (PMC7847196; doi:10.7717/peerj.10681)
Supplement: Table S1 [file peerj-09-10681-s007.docx]

| a (mm) | | b (mm/day) | | E |
| --- | --- | --- | --- | --- |
| Mean  (%RSE) | Variance  (%RSE) | Mean  (%RSE) | Variance  (%RSE) | Variance  (%RSE) |
| 3.82  (0.51) | 0.005  (12.00) | 0.08  (5.46) | 0.5633  (11.00) | 0.05  (2.90) |

RSE – relative standard error;
