# Supplementary material for: Exploring a model-based analysis of patient derived xenograft studies in oncology drug development
Source: PeerJ. 2021 Jan 27;9:e10681. doi: 10.7717/peerj.10681 (PMC7847196; doi:10.7717/peerj.10681)
Supplement: Table S3 [file peerj-09-10681-s009.docx]

| **Treatment** | **Day** | **TGI** | **Treatment Effect (c)** |
| --- | --- | --- | --- |
| BYL719 + HSP990 | 14 | 116 | 0.0383 |
|  | 21 | 75.5 | 0.0163 |
|  | 28 | 41.5 | -0.0282 |
| BYL719 | 14 | 67.6 | 0.0115 |
|  | 21 | 42.7 | -0.0355 |
|  | 28 | 88.4 | 0.0244 |
| HSP990 | 14 | 52.9 | -0.0292 |
|  | 21 | 69.5 | -0.00337 |
|  | 28 | 66.6 | -0.0163 |
| LEE011 | 14 | 68.7 | 0.00445 |
|  | 21 | 49.7 | -0.0107 |
|  | 28 | 98 | 0.0447 |
| LJM716 | 14 | 59.9 | 0.0033 |
|  | 21 | 94.5 | 0.0331 |
|  | 28 | 44.6 | -0.0225 |
| everolimus | 14 | 109 | 0.062 |
|  | 21 | -2.13 | -0.061 |
|  | 28 | 84.7 | 0.0325 |
| BYL719 + LJM716 | 14 | 88.2 | 0.0408 |
|  | 21 | 75.9 | 0.0106 |
|  | 28 | 69.6 | -0.00222 |
| LEE011 + everolimus | 14 | 94.9 | 0.0321 |
|  | 21 | 82 | 0.0315 |
|  | 28 | 95.7 | 0.0308 |
| BKM120 | 14 | 77.5 | 0.0231 |
|  | 21 | 110 | 0.0459 |
|  | 28 | 67.1 | -0.00253 |
| BKM120 + LJC049 | 14 | 38.9 | -0.019 |
|  | 21 | -21.1 | -0.0675 |
|  | 28 | 87.8 | 0.0221 |
| BYL719 + binimetinib | 14 | 112 | 0.0798 |
|  | 21 | 107 | 0.0436 |
|  | 28 | 38.3 | -0.0311 |
| BYL719 + encorafenib | 14 | 82.4 | 0.0224 |
|  | 21 | 5.11 | -0.0326 |
|  | 28 | 18.1 | -0.0484 |
| CGM097 | 14 | 31.4 | -0.0442 |
|  | 21 | 35.5 | -0.0182 |
|  | 28 | 55.4 | -0.02 |
| CKX620 | 14 | 98.3 | 0.0504 |
|  | 21 | 27 | -0.0247 |
|  | 28 | 68.8 | -0.00182 |
| HDM201 | 14 | 38.5 | -0.0234 |
|  | 21 | 37.2 | -0.0233 |
|  | 28 | 79.9 | 0.0143 |
| cetuximab | 14 | 59.2 | -0.012 |
|  | 21 | 82.7 | 0.0164 |
|  | 28 | 71.8 | 0.0126 |
| encorafenib | 14 | 41.3 | -0.027 |
|  | 21 | 82.6 | 0.017 |
|  | 28 | 30.6 | -0.031 |
| LJC049 | 14 | 7.94 | -0.0503 |
|  | 21 | 53.5 | -0.0423 |
|  | 28 | 40.2 | -0.0106 |
| binimetinib | 14 | 74.4 | 0.0179 |
|  | 21 | 85.5 | 0.0145 |
|  | 28 | 42.9 | -0.0266 |
| LFW527 + binimetinib | 14 | -4.99 | 0.0361 |
|  | 21 | 21 | -0.0413 |
|  | 28 | 48.4 | -0.0246 |
| CLR457 | 14 | 85.1 | 0.0228 |
|  | 21 | 62 | -0.00997 |
|  | 28 | 11.4 | -0.0398 |
| figitumumab" | 14 | 53.1 | -0.0381 |
|  | 21 | 46.8 | -0.0356 |
|  | 28 | 6.83 | -0.0517 |
| INC280 | 14 | 27.3 | -0.0378 |
|  | 21 | 104 | 0.0423 |
|  | 28 | 98.1 | 0.0292 |
| INC280 + trastuzumab | 14 | 55.8 | -0.0543 |
|  | 21 | 50.7 | -0.0212 |
|  | 28 | 88.1 | 0.0246 |
| LJM716 + trastuzumab | 14 | 30.9 | -0.0249 |
|  | 21 | -55.9 | -0.0746 |
|  | 28 | 49.4 | -0.0232 |
| LLM871 | 14 | 8.09 | -0.0423 |
|  | 21 | 83.5 | 0.00474 |
|  | 28 | 59.7 | -0.0119 |
| trastuzumab | 14 | 36.4 | -0.0449 |
|  | 21 | 64 | 0.0434 |
|  | 28 | 37.2 | -0.0317 |
| INC424 | 14 | 60.9 | -0.0151 |
|  | 21 | 23.5 | -0.0175 |
|  | 28 | 19 | -0.0257 |
| LFA102 | 14 | -113 | -0.0568 |
|  | 21 | 36.3 | -0.0319 |
|  | 28 | 104 | 0.0463 |
| LKA136 | 14 | 43 | -0.0297 |
|  | 21 | 94.4 | 0.0356 |
|  | 28 | 60.7 | -0.0115 |
| paclitaxel | 14 | 63 | -0.00741 |
|  | 21 | 71.3 | 0.0263 |
|  | 28 | 68.3 | 0.0207 |
| LFW527 + everolimus | 14 | 96.2 | 0.0392 |
|  | 21 | 9.25 | -0.0362 |
|  | 28 | 41 | 0.00848 |
| BYL719 + cetuximab | 14 | 69.4 | 0.00196 |
|  | 21 | -27.4 | -0.0553 |
|  | 28 | 94.7 | 0.0276 |
| BGJ398 | 14 | 40.9 | -0.0349 |
|  | 21 | 15.4 | -0.0549 |
|  | 28 | -27.9 | -0.0474 |
| erlotinib | 14 | 89.9 | 0.0818 |
|  | 21 | 26.7 | -0.0439 |
|  | 28 | 90.1 | 0.0388 |
| LCL161 + paclitaxel | 14 | 104 | 0.0733 |
|  | 21 | 27.1 | -0.0426 |
|  | 28 | 99.5 | 0.0632 |
| LGH447 | 14 | 38 | -0.0207 |
|  | 21 | 107 | 0.0513 |
|  | 28 | 42.1 | -0.04 |
| BKM120 + binimetinib | 14 | 103 | 0.0329 |
|  | 21 | 64.1 | -0.00704 |
|  | 28 | 79.7 | 0.0129 |
| BKM120 + LDE225 | 14 | 94.1 | 0.0343 |
|  | 21 | 104 | 0.0325 |
|  | 28 | 43.1 | -0.0288 |
| gemcitabine-50mpk | 14 | 54 | -0.0203 |
|  | 21 | 78.4 | 0.0349 |
|  | 28 | 84 | 0.0255 |
| INC424 + binimetinib | 14 | 93 | 0.0165 |
|  | 21 | 105 | 0.0686 |
|  | 28 | 40.6 | 0.0243 |
| trametinib | 14 | 80.7 | 0.00666 |
|  | 21 | 94.4 | 0.0431 |
|  | 28 | 2.6 | -0.046 |
| BYL719 + LEE011 | 14 | 100 | 0.0521 |
|  | 21 | 57.1 | 0.0267 |
|  | 28 | 35.5 | -0.0384 |
| tamoxifen | 14 | -7.22 | -0.0662 |
|  | 21 | 59.9 | -0.0189 |
|  | 28 | 48 | -0.00495 |
| BYL719 + cetuximab + encorafenib | 14 | 63.7 | 0.0108 |
|  | 21 | 89.4 | 0.00389 |
|  | 28 | 75.1 | 0.0363 |
| cetuximab + encorafenib | 14 | 2.85 | -0.0602 |
|  | 21 | 58.7 | -0.0161 |
|  | 28 | 107 | 0.0476 |
| BYL719 + LGH447 | 14 | 44.8 | 0.00376 |
|  | 21 | 87.6 | 0.0201 |
|  | 28 | 98.1 | 0.0309 |
| BKM120 + encorafenib | 14 | 99.9 | 0.0884 |
|  | 21 | 57.3 | -0.0212 |
|  | 28 | 81.4 | -0.0105 |
| LDK378 | 14 | 43.2 | -0.0161 |
|  | 21 | -225 | -0.00383 |
|  | 28 | 100 | 0.0409 |
| WNT974 | 14 | -91.8 | -0.0398 |
|  | 21 | 77.5 | 0.0226 |
|  | 28 | 59.9 | 0.0109 |
| TAS266 | 14 | 59.8 | 0.0236 |
|  | 21 | 41.2 | -0.0474 |
|  | 28 | 28.4 | -0.0584 |
| LEE011 + encorafenib | 14 | 112 | 0.0672 |
|  | 21 | 83.9 | 0.0342 |
|  | 28 | 64 | 0.0414 |
| encorafenib + binimetinib | 14 | 62.6 | -0.0142 |
|  | 21 | 64.7 | 0.0588 |
|  | 28 | 44.2 | -0.0293 |
| figitumumab" + binimetinib | 14 | 48.8 | -0.0335 |
|  | 21 | 87.7 | 0.0374 |
|  | 28 | 59 | -0.00239 |
| binimetinib-3.5mpk | 14 | 62 | -0.00914 |
|  | 21 | 77.9 | 0.0487 |
|  | 28 | -12.1 | -0.0619 |
